# Supplementary material for: Benchmarking Spectral Library and Database Search Approaches for Metaproteomics Using a Ground-Truth Microbiome Dataset
Source: bioRxiv. 2025 May 20:2025.05.15.654320. Preprint. [Version 1] doi: 10.1101/2025.05.15.654320 (PMC12139738; doi:10.1101/2025.05.15.654320)
Supplement: Supplement 1 [file media-1.pdf]

Supplemental Information for Benchmarking Spectral Library and Database Search Approaches for Metaproteomics Using a Ground-Truth Microbiome Dataset

Andrew T. Rajczewski[1]+, Subina Mehta[1]+, Reid Wagner[1], Wassim Gabriel[2], James Johnson[1], Katherine Do[1], Simina Vintila[4], Mathias Wilhelm[2,3], Manuel Kleiner[4], Brian C. Searle[5], Timothy J. Griffin[1], Pratik D. Jagtap[1]*.

1. University of Minnesota, Minneapolis, MN
2. Computational Mass Spectrometry, Technical University of Munich, Freising, Germany
3. Munich Data Science Institute, Technical University of Munich, Garching, Germany
4. North Carolina State University, Raleigh, NC
5. Department of Quantitative Health Sciences, Mayo Clinic, Rochester, MN

+ Contributed equally
** Corresponding author*

Supplementary Table 1: Protein sequence database components used in the proteomic analyses of composite microbiome samples

**Label**	**Species**	**Input Protein Amount (ug)**	**Percentage**
Ne1	*Nitrosomonas europaeae*	60.58	0.082
F2	Phage F2	62.04	0.084
F0	Phage F0	65.28	0.088
ES18	Phage ES18	65.55	0.088
P22	Phage P22	78.77	0.106
M13	Phage M13	109.02	0.147
Nm1	*Nitrosospira multiformis*	155.16	0.209
BXL	*Burkholderia xenovorans*	321.37	0.433
Nu1	*Nitrosomonas ureae*	402.68	0.543
BS	*Bacillus subtilis*	583.83	0.788
NV	*Nitrososphaera viennensis*	607.41	0.819
841	*Rhizobium leguminosarum bv. viciae* 3841	680.47	0.918
PaD	*Paracoccus denitrificans*	683.66	0.922
DVH	*Desulfovibrio vulgaris*	701.58	0.946
Am2	*Alteromonas macleodii*	707.47	0.954
137	*Staphylococcus aureus* ATCC 13709	715.15	0.965
KF7	*Pseudomonas pseudoalcaligenes*	863.80	1.165
CV	*Chromobacterium violaceum*	933.08	1.259
AK199	*Roseobacter sp.* AK199	1183.43	1.596
259	*Staphylococcus aureus* ATCC 25923	1216.31	1.641
HB2	*Thermus Thermophilus*	1245.63	1.680
VF	*Rhizobium leguminosarum bv. viciae* VF39	1671.03	2.254

PD	*Pseudomonas denitrificans*	2128.51	2.871
CRH	*Chlamydomonas reinhardtii*	2962.73	3.996
ATN	*Agrobacterium tumefaciens*	4186.28	5.647
K12	*Escherichia coli*	4290.54	5.788
Pfl	*Pseudomonas fluorescens*	4964.22	6.696
SMS	*Stenotrophomonas maltophilia*	5946.27	8.021
Cup	*Cupriavidus metallidurans*	11504.98	15.519
LT2	*Salmonella enterica typhimurium* (3 strains combined)	25037.78	33.773

The bubble plot (**Figure 1**) gives an overview of the organisms detected from all of the searches. Notably, the low abundance proteins associated with Phage ES18 are detected only by Scribe and not by any other search algorithm. However, peptide from Phage ES18 could not be confirmed using PepQuery. PepQuery analysis of the peptides associated with the phages confirmed the presence of F0 peptide (EVESITPDEIQG) and P22 peptides (NVLAQDATFSVVR and QVAGFDDVLR) in the mass spectrometry datasets. F0 peptide was detected using FragPipe and Scribe, while the P22 peptide (NVLAQDATFSVVR) was detected by all search algorithms and P22 peptide (QVAGFDDVLR) was detected by FragPipe.

Supplementary Table 2: Detection of phage peptides and their verification.

		MaxQuant		FragPipe		Scribe		PepQuery			
**Organism**	**Peptide**	**1X**	**2X**	**1X**	**2X**	**1X**	**2X**	**U1**	**U2**	**U3**	**U4**
**F0**	EVESITPDEIQGVR			**Yes**	**Yes**	**Yes**	**Yes**			**Yes**	
**ES18**	DQEVGMEIVNDLIGVQTVLPVGK					**Yes**	**Yes**				
**P22**	NVLAQDATFSVVR	**Yes**	**Yes**	**Yes**	**Yes**	**Yes**	**Yes**	**Yes**	**Yes**	**Yes**	
**P22**	YTPPAASMQR					**Yes**	**Yes**				
**P22**	ADDLRDETAYR			**Yes**	**Yes**						
**P22**	QVAGFDDVLR			**Yes**							**Yes**
**P22**	ALNEGQIVTLAVDEIIETISAITPMAQK			**Yes**	**Yes**						
**P22**	TTSFSIPDVGLNGIFATQGDISTLSGLCR			**Yes**	**Yes**						
**P22**	VVDGTHVEITPKPVALDDVSLSPEQR			**Yes**	**Yes**						

A

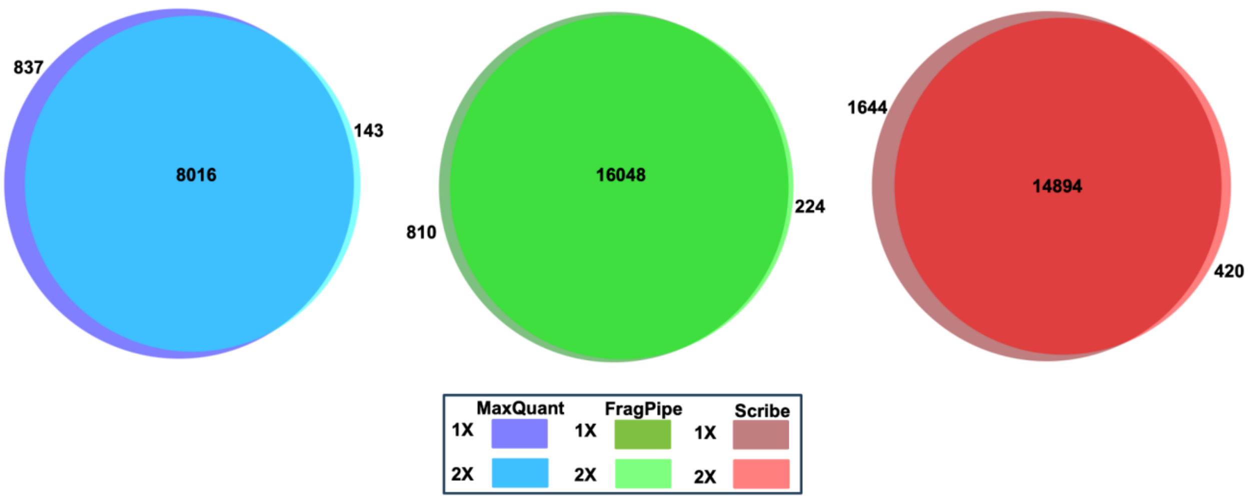

B

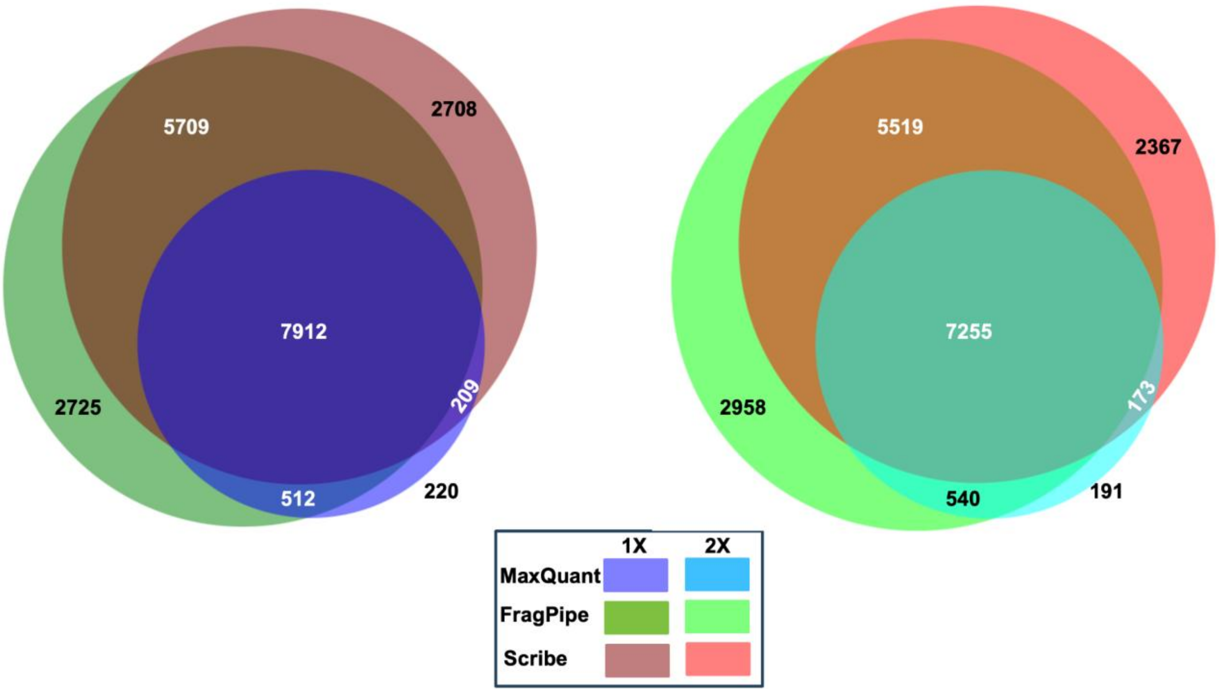

Supplementary Figure 1. Overlap of peptides detected from the synthetic microbiome database. A) Peptides detected by MaxQuant, FragPipe, and Scribe have been represented. The color scheme for the searches against databases of variable sizes. **B)** Peptides detected by 1X and 2X database size by the three search algorithms have been represented. The color scheme for the searches against databases of variable sizes.

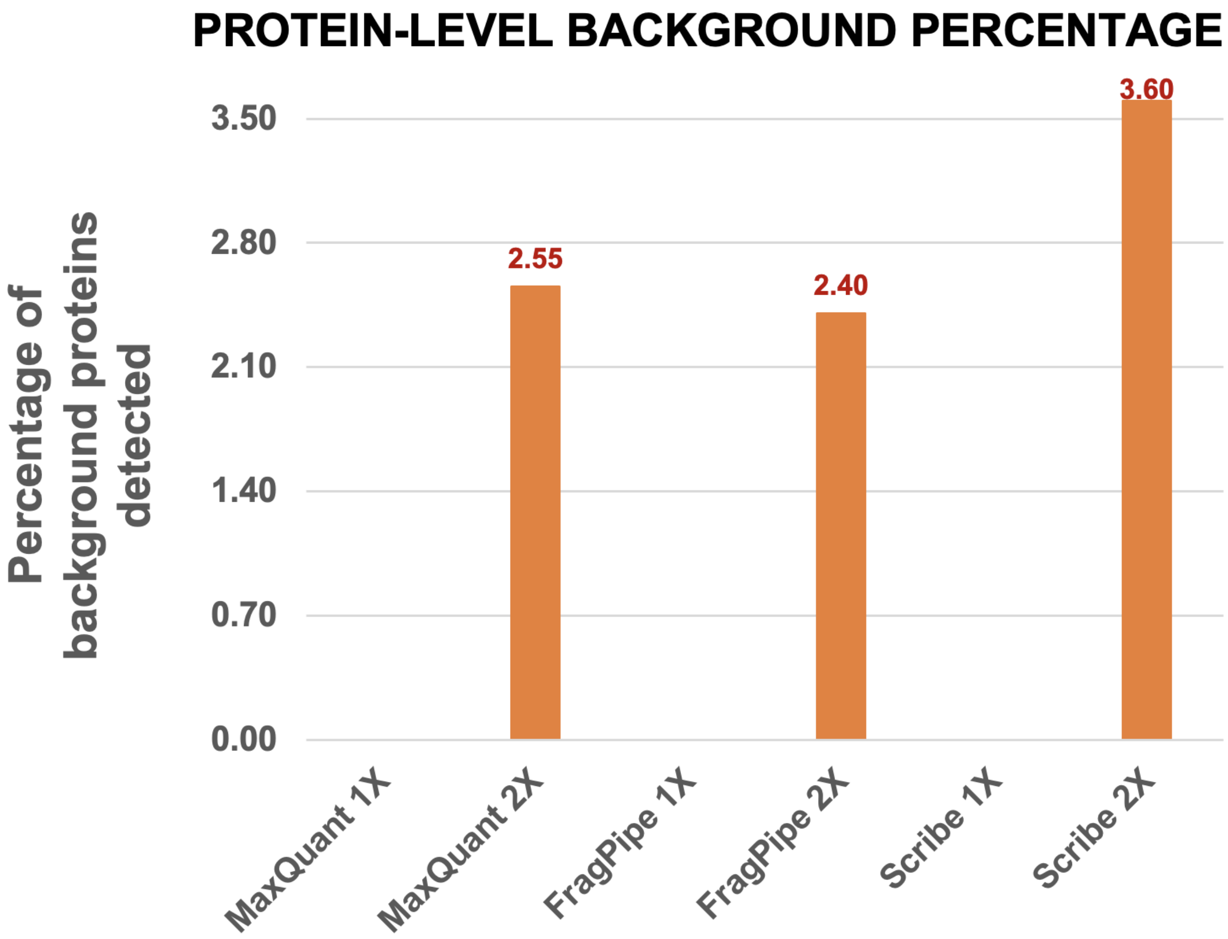

Supplementary Figure 2. Percentage of background proteins detected in MaxQuant, FragPipe and Scribe searches. The percentage was calculated from protein-level outputs (Figure 2) from the three search algorithms.

For background proteins detected, FragPipe showed the best performance with the percent of background proteins detected at 2.4% (for 2X database search). As compared to this, MaxQuant detected 2.55% of background proteins (for 2X database) and Scribe detected 3.6% of background proteins (for 2X database), respectively.

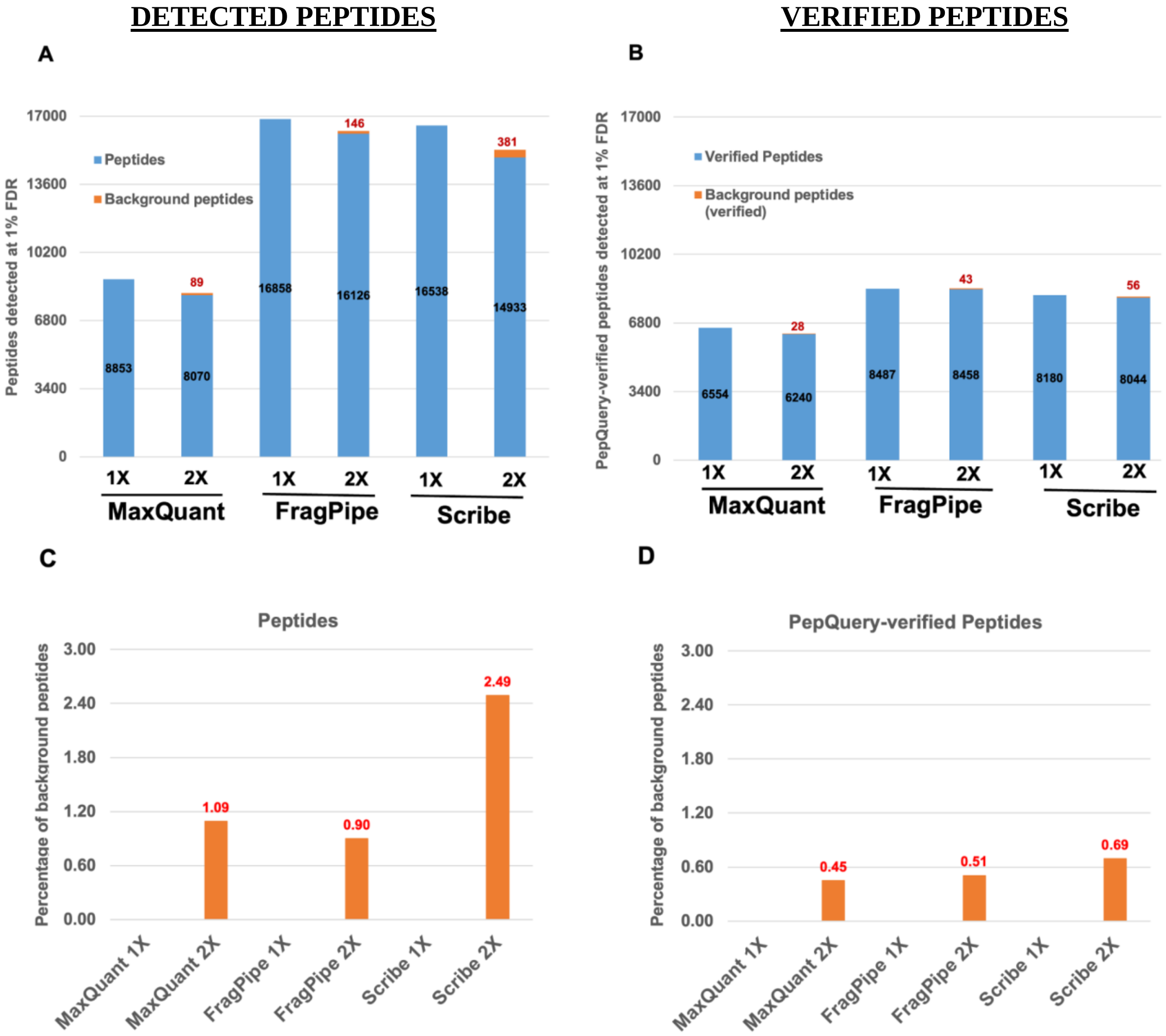

Supplementary Figure 3. Peptides detected from the synthetic microbiome database and the background (IGC database). Peptides detected at 1%FDR by MaxQuant, FragPipe and Scribe have been represented in the top left panel. The top left panel figure (A) is the same as Figure 2B in main text and has been shown to facilitate comparison with the figure in the top right panel (B), which shows detected peptides after data processing using PepQuery. The peptides detected from the synthetic microbiome database have been shown in blue, while background peptides have been shown in red. Bottom left panel (C) shows the percentage of background peptides detected in MaxQuant, FragPipe and Scribe searches. The percentage was calculated from peptide-level outputs from the top left panel. Bottom right panel (D) shows the percentage of background peptides (verified) processed after MaxQuant, FragPipe and Scribe searches. The percentage was calculated from peptide numbers from the top right panel.

We investigated the nature of the background peptides detected. After looking at the search results carefully, we found that some of the background peptides were shared with peptides from the 30-organism database. After PepQuery analysis of all the peptides against the 30-microorganisms database, the background peptide detection rate for MaxQuant improved from 1.1% to 0.45% (**Supplementary Figure 3C and 3D**), and the background peptide detection rate for FragPipe improved from 0.9% to

0.51%. Scribe, which had the highest background peptide detection rate of 2.49% showed an improvement to an impressive 0.69% after PepQuery analysis.

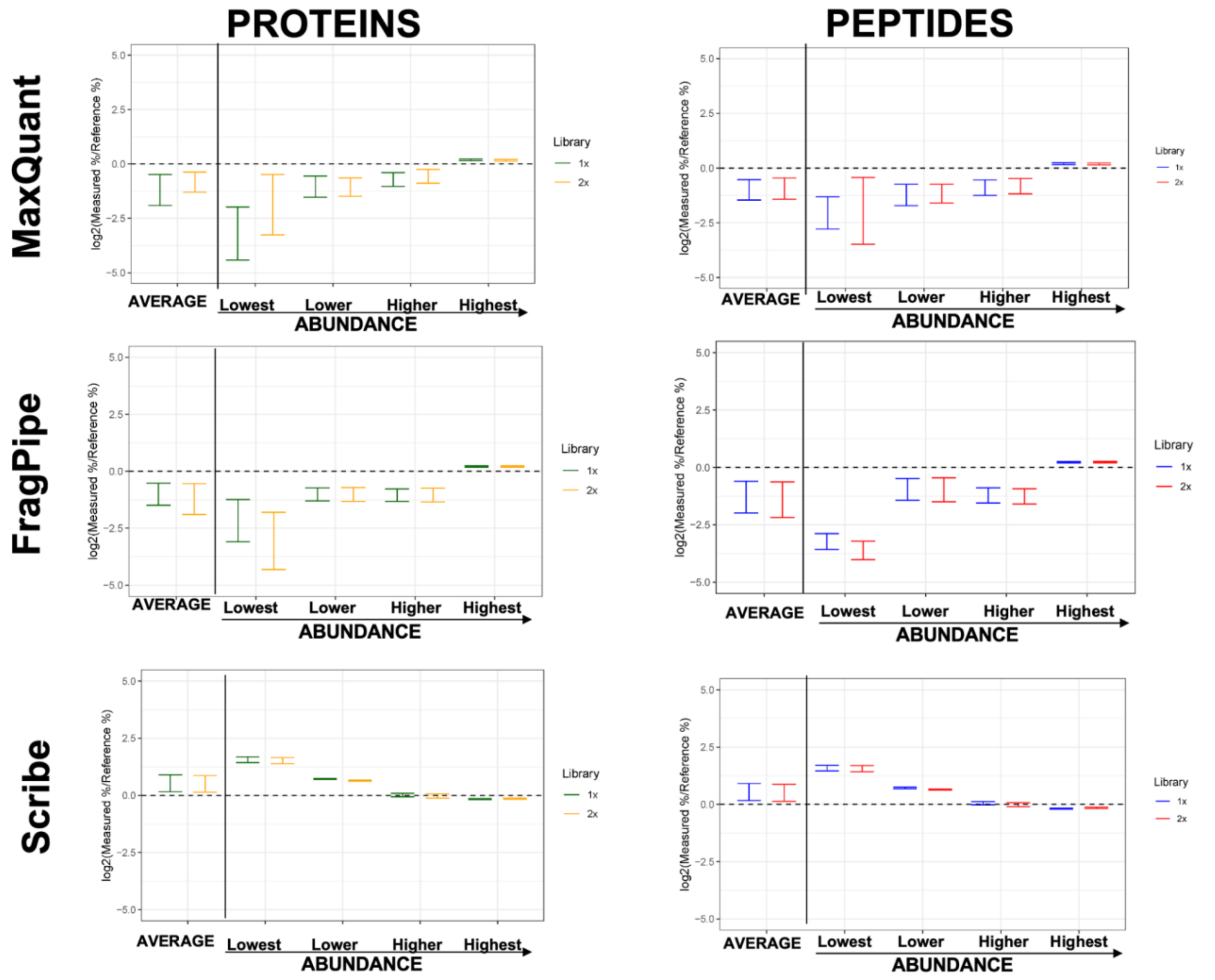

Supplementary Figure 4. Quantitative analyses of the search results demonstrate a higher correlation between measured and reference values in the Scribe platform. The protein-level and peptide-level intensities of the organisms of lowest abundance (Ne1, F0, ES18, P22, Nm1, BXL and Nu1); lower abundance (BS, NV, 841, PaD, DVH, Am2 and 137); higher abundance (KF7, CV, AK199, 259, HB2, VF, and PD) and highest abundance (CRH, ATN, K12, Pfl, SMS, Cup and LT2) were summed up. The percentage of the summed intensity at protein-level or peptide-level were compared to the expected percentage of the various tiers of organisms. The line at 0.0 indicates absence of any difference between expected and measured values.
